# Dietary habits among men and women in West Greenland: follow-up on the ACCEPT birth cohort

**DOI:** 10.1186/s12889-021-11359-7

**Published:** 2021-07-19

**Authors:** Maria Wielsøe, Dina Berthelsen, Gert Mulvad, Silvia Isidor, Manhai Long, Eva Cecilie Bonefeld-Jørgensen

**Affiliations:** 1 Department of Public Health, Aarhus University, Centre for Arctic Health & Molecular Epidemiology, Aarhus, Denmark; 2 Health Care, Queen Ingrid’s Health Centre, Nuuk, Greenland; 3 Greenland Centre for Health Research, University of Greenland, Nuuk, Greenland

**Keywords:** Diet, Traditional food, Country food, Imported food, Arctic, Greenland

## Abstract

**Background:**

In the past decades, the diet in Greenland has been in transition resulting in a lower intake of traditional food and a higher intake of imported western food. This diet transition can affect public health negatively, and thus, continued monitoring of dietary habits is important. The present study aimed to follow up on the dietary habits of pregnant women included in the Greenlandic ACCEPT birth cohort (2013–2015) and the children’s father.

**Methods:**

The follow-up food intake was assessed in 2019–2020 using food frequency questionnaires for 101 mothers and 76 fathers aged 24–55 years living in Nuuk, Sisimiut, and Ilulissat. Non-parametric statistical methods were used (Mann-Whitney U test/Spearman correlation) to assess the dietary pattern and influencing factors.

**Results:**

The proportion of traditional and imported food was 14 and 86%, respectively. Intake frequency differed by gender (vegetables, fruits, fast food), the living town (terrestrial animals, vegetables, fruits), and age (fish, meat products, fruits, fast food). Socioeconomic and lifestyle factors significantly correlated with the intake frequency of several traditional and imported foods. Few changes in the mother’s dietary habits from inclusion (during pregnancy) to follow-up (3–5 years later) were found, showing less frequent intake of seabirds and fruits and more frequent meat intake.

**Conclusion:**

We identified several factors that could affect dietary habits, and the results may be used to target future food recommendation for relevant population groups.

**Supplementary Information:**

The online version contains supplementary material available at 10.1186/s12889-021-11359-7.

## Background

People have inhabited Greenland for at least 4500 years and have traditionally had a hunting-based lifestyle and diet [1]. Marine mammals (seal, whale, walrus, and polar bear), seabirds, and fish have traditionally dominated the diet of Greenlandic Inuit, while roots, berries, and leaves were eaten as a supplement when in season. Since the beginning of the twentieth century, the Greenlandic population has experienced a transition towards a society more influenced by the Western world [2, 3]. This transition has included a change towards a more Western diet. The diet today includes many imported food items, such as meat products from farm animals, fruit, vegetables, dairy products, and sugary foods. Despite the transition away from the traditional Greenlandic diet, it remains an important cultural, spiritual, and social connection to the surrounding nature [3].

The traditional Greenlandic diet, including marine mammals and fish, is an essential contributor to the intake of long-chain omega-3 fatty acids (n-3), minerals/metals (Iron, Zink, Selenium, Iodine), and vitamin A, B, and D [4]; however, the level of dietary fibers and other vitamins such as vitamin C are low [5]. Several of the nutrients in the traditional diet have been suggested to have a positive impact on human health, such as protective effects against inflammation [6, 7] and cardiovascular disease [8, 9], together with beneficial effects on brain and nerve development in children [10, 11]. However, the marine mammals, on top of the food chain, also contain high levels of contaminants, such as persistent organic pollutants (POPs) and toxic metals [12]. These environmental contaminants have been associated with several negative health effects, including impaired fetal growth [13, 14], adverse effects on the immune-, neuro-, reproductive, and endocrine system, and increased risk of some cancers [12].

The imported food products are often energy-dense and can be nutrient-poor products, which have a long shelf life and some are low-priced, available in all places including remote settlements [15]. Some of the popular imported food items include carbonated sweets, chips, and farmed (red) meat with a high saturated fat content. Together with a decrease in physical activity, the dietary transition has contributed to the increasing incidence of overweight, obesity, and diabetes in Greenland [15].

In Greenland, the Population Health Surveys showed a reduction in intake of local traditional Greenlandic food items from 2005 to 2018 and an increased intake of imported meat, fruit, vegetables, and dairy products [16, 17]. In 2005–2010, the proportion of traditional food was 17% in towns and 32% in settlements, while in 2018, the traditional food intake decreased to 14 and 21%, respectively [17]. In 2005–2010, the Nordic Nutrition Recommendations for intake of added sugar were exceeded by 67–71% of the participants, for saturated fat by 39–44%, and for protein by 46–47%, while 77% were below the recommendation for fibre and 47–59% were below for carbohydrate [5].

Dietary habits were also assessed in the Greenlandic birth cohort ACCEPT (Adaptation to Climate Change, Environmental Pollution, and dietary Transition), which included pregnant women from all parts of Greenland in 2010–2015 [18, 19]. The proportion of traditional food among pregnant women was 12–18% [18, 19], thus comparable to the proportions found in the general population in the Population Health Surveys [16, 17]. The most frequently consumed traditional food groups were marine mammals and fish, while the most frequently consumed imported food groups were fruit, carbohydrate food (potato, pasta, and rice), and sweets and snacks [18, 19].

Dietary habits can affect health and the risk of diseases, including diabetes, heart disease, stroke, and cancer [20]. Foods related to a reduced disease risk include fish, whole grain products, fruit, and vegetables, and these are often referred to as healthy foods. Whereas intake of processed and red meat, sugary snacks and sweets, fast food, and sugary drinks can increase the risk of some diseases and could be referred to as unhealthy foods. However, the amount eaten and the total energy intake play an important role as well. Thus, one of the most important food recommendations is to eat a varied, well-balanced, and healthy diet [20]. The health implications of the ongoing dietary transition in Greenland are complex, as both traditional and imported food can positively and negatively affect health. The transition must be followed closely to be able to revise and update the food recommendations continuously.

From May 2019 to January 2020, we followed up 101 of the women included in the ACCEPT birth cohort from 2013 to 2015, fathers of the children, and the ACCEPT children age 3–5 years. The study presents dietary patterns of the father and mother (aged 24–55 years) with possible differences for gender, age, and living place. We also looked into possible correlations in dietary habits between parents living together. To our knowledge, this is the first time in Greenland, that longitudinal dietary changes are reported, as we report dietary changes for women from inclusion at pregnancy to follow-up (3–5 years later).

## Materials and methods

### Study population

**Fig. 1:**
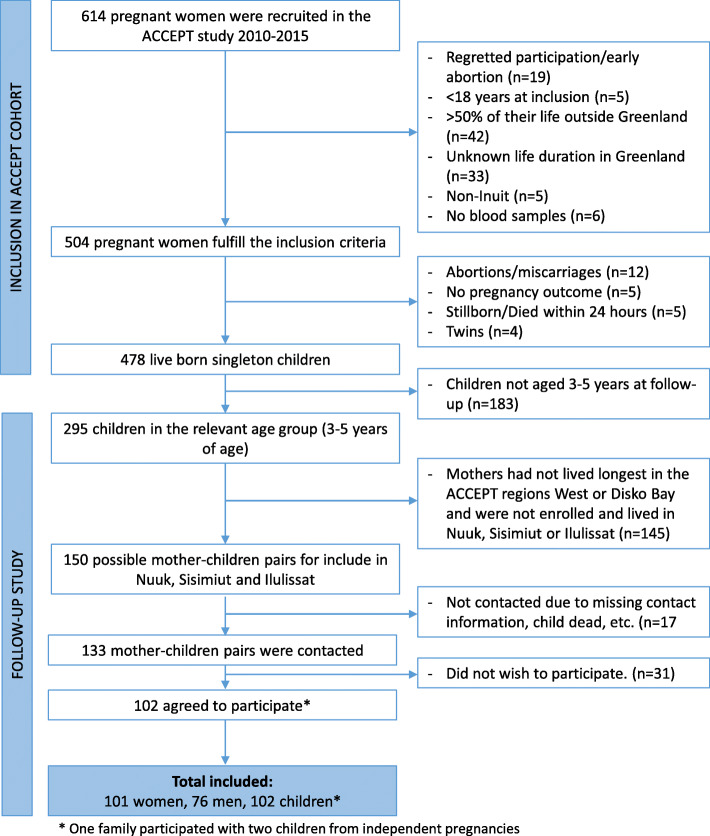
Flow chart for the study

This study is based on the ACCEPT birth cohort [18, 19] established during 2010–2015 in Greenland with the overall aim of exploring environmental exposures, dietary changes, and health during a period of lifestyle transition and climate change. In total, 614 Greenlandic pregnant women were recruited from 16 towns distributed over five regions of Greenland (North, Disko bay, West, South, and East). To be included in the ACCEPT study, the women had to be over 18 years of age at the time of inclusion, lived more than 50% of their lives in Greenland, and have at least one Inuit parent. Of the recruited ACCEPT women, 504 fulfilled the inclusion criteria and 478 completed their pregnancy with available birth outcome (Fig. 1).

During this ACCEPT follow-up study (May 2019 to January 2020), 295 live born singleton children were in the relevant age group of 3–5 years of age (children of mothers recruited in 2013–2015). Of these, 150 fulfilled the follow-up inclusion criteria (mothers had lived longest in the ACCEPT regions West or Disko Bay and currently lived in Nuuk, Sisimiut, or Ilulissat). The child’s biological father was included in this follow-up if possible. We contacted 133 ACCEPT mothers and her partner/child father, and 102 agreed to participate (one family participated with two children from two independent pregnancies). Finally, the total study population at follow-up included 101 mothers, 76 fathers, and 102 ACCEPT children (Fig. 1). The participation rate at follow-up was 76.6%, and those who did not accept to participate mainly gave lack of time as the reason.

After receiving a detailed description of the study, all participants gave signed informed consent to participate. They were informed that they could withdraw their consent at any time in the process and that their participation was voluntary.

The families got two home visits from a health nurse visitor and project researchers, respectively. At the first visit, the health nurse visitor interviewed the families, and questionnaires were filled out, and at the second visit, biological samples (blood, hair, urine, and nails) were collected.

The study was carried out in accordance with the Helsinki convention II, and was approved by the Commission for Scientific Investigations in Greenland (KVUG 2019–04).

### Questionnaire data

The adult participants completed a self-administered questionnaire in Danish or Greenlandic. The health nurse visitors were available for assistance if the participants were in doubt about the meaning of the questions and possible answers. Two independent person’s double-entered data from the questionnaire into the validation program EpiData, and any disagreements were discussed.

The adult participants’ questionnaire was divided into two sections; section 1 contained questions about demographics, lifestyle, and health, and section 2 was a food frequency questionnaire (FFQ) about food intake for the last 12 months.

Data extracted from section 1 included age, history of living places (in and outside of Greenland), ethnicity, educational level, income, alcohol intake, smoking history, drug use, body mass index (BMI) (from self-reported height and weight), and number of children.

The dietary intake was calculated based on an FFQ (section 2 of the questionnaire), which was a slightly optimized version of the questionnaire used at inclusion at pregnancy during 2013–2015 [19]. It contained questions about 42 traditional and 23 imported food items, and it was possible to report the consumption frequencies in eight categories ranging from “never” to “several times a day” for each food item. The food items were divided into seven main traditional food groups: Marine mammals, Seabirds, Fish, Dried fish, Shellfish, Terrestrial animals, and Berries and seven main imported food groups: Meat products, Carbohydrate foods, Sauce, Fruit, Vegetables, Fast food, and Sweets and Snacks (Additional file 1: Table S1A). Each food item was given a frequency score representing the time(s) a month the food item was consumed. The food consumption frequencies for the food groups were calculated by summing the scores for the specific food items in the group. See additional file 1 for further explanation and examples of the calculation (Additional file 1: Table S1B, S1C). The frequency score method and similar FFQs have been used previously [18, 19, 21–23].

Furthermore, the FFQ included a column for seasonal information, with the possibility to answer if the food item was consumed in the spring/summer and/or autumn/winter. However, the percentage of missing data in these season variables was high (up to 60%), and the information was not included in the analyses. Please see Additional file 2: Table S2B.

### Statistics

All statistical analyses were performed with SPSS software version 27 (SPSS Inc., Chicago, IL, USA). The statistically significant level was set to *p* ≤ 0.05.

For continuous variables, Mann-Whitney U test was used to test the difference between groups due to a high degree of non-normal distributed data (assessed by Q-Q plots) for the food variables. For categorical variables, Pearson’s Chi-square test was used to test the difference.

Correlations between intake frequency of food groups and socioeconomic and lifestyle factors were assessed with Spearman correlations.

The analyses of parent differences were restricted to mothers and fathers living together at the time of follow-up (*n* = 73). To assess the food intake differences between the individual parents, we subtracted the reported intake of the father from the reported intake of the mother (mother – father), thus zero (0) reflect equal intake for the mother and father, negative values reflect higher intake for the father, and positive values reflect higher intake for the mother.

To assess changes in food intake from inclusion at pregnancy to follow-up for the mother, the individual differences were calculated by subtraction of the reported intake from inclusion at pregnancy from the reported intake at follow-up (follow-up - inclusion). Thus zero (0) reflect equal intake at inclusion and follow-up, negative values reflect higher intake at inclusion during pregnancy, and positive values reflect higher intake at follow-up.

Wilcoxon Signed Rank test (median equal to 0) was used to test parent differences and changes from inclusion at pregnancy to follow-up (for mothers only) in food intake frequency. Furthermore, we assessed the pairwise correlations by calculating Spearman correlations for the food intake frequency. We also calculated partial Spearman correlations by adjusting for different predictors of the food intake frequency. The correlations between the parents were adjusted for the age difference between the parents (continuous), household income (categorical), percentage of life lived in Greenland for both the father and mother (continuous), and highest educational level for both the father and mother (categorical). Whereas, the correlations between inclusion at pregnancy and follow-up of the mother were adjusted for the time from inclusion to follow-up (continuous), change in education level (categorical), change in household income (categorical), change in BMI (continuous), and change in parity (categorical).

Sensitivity analyses by excluding non-Inuit and persons with less than 50% of their life spent in Greenland were conducted.

## Results

### Characteristics

Table 1presents the characteristics of the participants in the present study (101 mothers/ 76 fathers). The mothers were younger and leaner compared to the fathers. The mothers were also more likely to have a university degree and have never used hash and drugs, but less likely to have a personal income of more than 250.000 DKK per year.

**Table 1:** Characteristics of the ACCEPT follow-up study population

		Mothers (***N*** = 101)	Fathers (***N*** = 76)	*p*-value^**a**^	All (*N* = 177)
**Age (years)**					
	Mean (SD)	33.8 (4.8)	37.2 (7.5)	**0.005**^b^*	35.3 (6.3)
	Median (P25–P75)	34.0 (29.8–37.3)	36.7 (30.8–42.2)		34.3 (30.3–39.2)
	Missing n (%)	0 (0%)	0 (0%)		0 (0%)
**Current living town**					
Nuuk	n (%)	69 (68.3%)	50 (65.8%)	0.723^c^	119 (67.2%)
Other towns^d^	n (%)	32 (31.7%)	26 (34.2%)		58 (32.8%)
	Missing n (%)	0 (0%)	0 (0%)		0 (0%)
**Percentage of life lived in Greenland (%)**	Mean (SD)	95.0 (6.8)	89.7 (20.1)	0.503^b^	92.7 (14.3)
	Median (P25–P75)	97.5 (93.6–99.2)	97.8 (91.1–98.8)		97.5 (93.5–99.0)
	Missing n (%)	1 (1%)	1 (1%)		2 (1%)
**Region lived longest**					
Disko bay	n (%)	25 (26.6%)	13 (18.6%)	**< 0.001**^c^*	38 (23.2%)
West	n (%)	69 (73.4%)	44 (62.9%)		113 (68.9%)
Other region in Greenland^e^	n (%)	0 (0.0%)	8 (11.4%)		8 (4.9%)
Outside Greenland	n (%)	0 (0.0%)	5 (7.1%)		5 (3.0%)
	Missing n (%)	7 (7%)	6 (8%)		13 (7%)
**Ethnic background**					
Inuit (both parents born in Greenland)	n (%)	79 (79.8%)	61 (80.3%)	**0.021**^c^*	140 (80.0%)
Partly-Inuit (one parent born in Greenland)	n (%)	20 (20.2%)	10 (13.2%)		30 (17.1%)
Non-Inuit (no parents born in Greenland)	n (%)	0 (0.0%)	5 (6.6%)		5 (2.9%)
	Missing n (%)	2 (2%)	0 (0%)		2 (1%)
**BMI (kg/m**^**2**^**)**					
	Mean (SD)	28.1 (5.3)	29.3 (4.9)	**0.050**^b^*	28.6 (5.1)
	Median (P25–P75)	26.4 (24.6–30.8)	28.6 (26.1–32.3)		27.8 (25.3–31.6)
	Missing n (%)	21 (21%)	12 (16%)		33 (19%)
Underweight (< 18.5)	n (%)	0 (0.0%)	0 (0.0%)	0.203^c^	0 (0.0%)
Normal (18.5–24.9)	n (%)	22 (27.5%)	12 (18.8%)		24 (23.6%)
Overweight (25.0–29.9)	n (%)	35 (43.8%)	25 (39.1%)		60 (41.7%)
Obese (> 30.0)	n (%)	23 (28.7%)	27 (42.2%)		50 (34.7%)
**Performing physical activities/sports**					
Yes	n (%)	43 (43.0%)	26 (34.2%)	0.276^c^	69 (39.2%)
No	n (%)	57 (57.0%)	50 (65.8%)		107 (60.8%)
	Missing n (%)	1 (1%)	0 (0%)		1 (1%)
**Highest education level**^**f**^					
Primary school	n (%)	21 (21.2%)	16 (21.3%)	**0.006**^c^*	37 (21.3%)
High School	n (%)	8 (8.1%)	6 (8.0%)		14 (8.0%)
Technical college	n (%)	32 (32.3%)	41 (54.7%)		73 (42.0%)
University	n (%)	38 (38.4%)	12 (16.0%)		50 (28.7%)
	Missing n (%)	2 (2%)	1 (1%)		3 (2%)
**Personal income per year**					
Under 100.000 DKK	n (%)	15 (15.2%)	5 (6.8%)	**0.014**^c^*	20 (11.6%)
100.000–250.000 DKK	n (%)	20 (20.2%)	10 (13.5%)		30 (17.3%)
Over 250.000 DKK	n (%)	52 (52.5%)	56 (75.7%)		108 (62.4%)
Don’t know	n (%)	12 (12.1%)	3 (4.1%)		15 (8.7%)
	Missing n (%)	3 (3%)	1 (1%)		4 (2%)
**Household income per year**					
Under 100.000 DKK	n (%)	2 (2.0%)	2 (2.7%)	0.311^c^	4 (2.3%)
100.000–250.000 DKK	n (%)	9 (9.2%)	5 (6.7%)		14 (8.1%)
Over 250.000 DKK	n (%)	76 (77.6%)	65 (86.7%)		141 (81.5%)
Don’t know	n (%)	11 (11.2%)	3 (4.0%)		14 (8.1%)
	Missing n (%)	3 (3%)	1 (1%)		4 (2%)
**Current alcohol consumption**					
0 drinks/week	n (%)	34 (55.7%)	38 (53.5%)	0.298^c^	72 (54.5%)
1–7 drinks/week	n (%)	22 (36.1%)	20 (28.2%)		42 (31.8%)
≥ 8 drinks/week	n (%)	3 (4.9%)	5 (7.0%)		8 (6.1%)
Don’t know	n (%)	2 (3.3%)	8 (11.3%)		10 (7.6%)
	Missing n (%)	40 (40%)	5 (7%)		45 (25%)
**Smoking history**					
Never smoker	n (%)	49 (48.5%)	37 (48.7%)	0.507^c^	86 (48.6%)
Previous smoker	n (%)	33 (32.7%)	20 (26.3%)		53 (29.9%)
Current smoker	n (%)	19 (18.8%)	19 (25.0%)		38 (21.5%)
	Missing n (%)	0 (0%)	0 (0%)		0 (0%)
**Hash use ever**					
Have used smoked/consumed hash	n (%)	42 (42.0%)	45 (60.0%)	**0.050**^c^*	87 (49.7%)
Never used smoked/consumed hash	n (%)	56 (56.0%)	28 (37.3%)		84 (48.0%)
Don’t know	n (%)	2 (2.0%)	2 (2.7%)		4 (2.3%)
	Missing n (%)	1 (1%)	1 (1%)		2 (1%)
**Drug use ever**					
Have taken other drugs	n (%)	3 (3.1%)	17 (23.3%)	**< 0.001**^c^*	20 (11.8%)
Never taken other drugs	n (%)	92 (95.8%)	55 (75.3%)		147 (87.0%)
Don’t know	n (%)	1 (1.0%)	1 (1.4%)		2 (1.2%)
	Missing n (%)	5 (5%)	3 (4%)		8 (5%)
**Number of children**					
	Mean (SD)	2.4 (1.1)	2.5 (1.2)	0.880^b^	2.4 (1.1)
	Median (P25–P75)	2.0 (2.0–3.0)	2.0 (2.0–3.0)		2.0 (2.0–3.0)
	Missing n (%)	0 (0%)	0 (0%)		0 (0%)
1	n (%)	19 (18.8%)	12 (15.8%)	0.365^c^	31 (17.5%)
2–3	n (%)	69 (68.3%)	55 (72.4%)		124 (70.1%)
4 or more	n (%)	13 (12.9%)	9 (11.8%)		22 (12.4%)

^a^: *P*-value for statistical test between mothers and fathers; ^b^: Mann-Whitney test; ^c^: Chi-square test; ^d^: Other towns include Sisimiut (20 mothers/ 16 fathers), Ilulissat (11 mothers/ 8 fathers), Kangerlussuaq (1 mothers/ 1 fathers) and Qaqortoq (1 father); ^e^: Other region in Greenland include North, South and East; ^f^: Educational level was interpreted from personal description for 7 participants. Bold *p*-values and * indicate significant difference (p ≤ 0.05)

Most of the participants lived in Nuuk (67.2%), while 32.8% lived in the other towns (Sisimiut, Ilulissat, Kangerlussuaq, and Qaqortoq). Due to the follow-up criteria, all mothers had lived longest in either Disko Bay or West, while some fathers had lived longest in other regions (11.4%) or outside Greenland (7.1%). Concerning the ethnic background, 80.0% were Inuit, 17.1% partly-Inuit, and 2.9% non-Inuit. An inclusion criterion for participating in the ACCEPT birth cohort was that the pregnant women were Inuit or partly-Inuit; thus, all non-Inuit participants in the present study were fathers (Table 1).

The results presented here were conducted on the full study population; however, the conducted sensitivity analyses excluding non-Inuit and persons with less than 50% of their life spent in Greenland showed similar results (not shown).

### Food intake

**Fig. 2:**
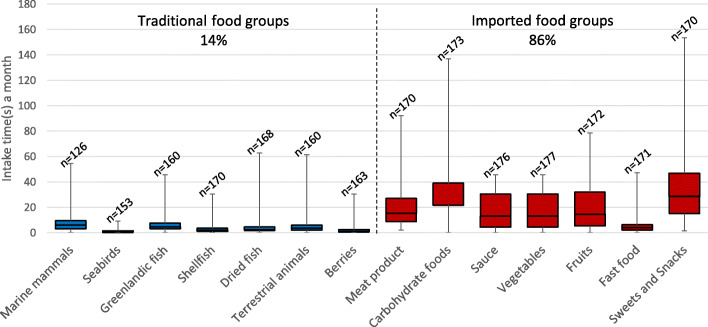
Traditional and imported food group intake (time(s) a month) (*N* = 177). The boxes display the 25th and 75th percentiles, and the line inside the boxes represents the median value. The whiskers display the minimum and maximum values. n: number of participants with information, The overall percentages of traditional (*x*) or imported food (*y*) food intake, were calculated by summing the medians of the main food groups and then the sum was divided by the total median intake (*x* + *y*)

Overall, the median intake frequency for traditional and imported food was distributed as 14 and 86%, respectively (Fig. 2 and Additional file 2, Table S2A). The most frequently consumed traditional food group was marine mammals (median: 6.0 times/month), followed by Greenlandic fish (median: 4.5 times/month) and terrestrial animals (median: 3.5 times/month), while the least frequently consumed traditional food groups were seabirds (median: 1.0 time/month) and berries (median: 1.0 time/month). The most consumed imported food group was carbohydrate food (median: 39.0 times/month) followed by sweets and snacks (median: 28.5 times/month), while fast food (median: 4.0 times/month) was the least consumed imported food group (Fig. 2 and Additional file 2: Table S2A). In Additional file 2 (Table S2B), the intake of the individual food items together with seasonal information can be seen. Of the traditional individual food items, caribou (wild-living Arctic reindeer) was most frequently consumed (median: 2.5 times/month), and 14.5% of the participants ate it at least once a week. For the imported food items, potato, pasta, rice, sauce, vegetables, and fresh fruit were all consumed 13.0 times/month (median), and 79.5–86.4% ate the food items at least once a week.

#### Gender differences

For all traditional food groups, the intake frequency was similar for mothers and fathers (Table 2). However, for the imported food groups, mothers reported more frequently consuming fruit and vegetables and less frequently fast food than the fathers. Similarly, a higher percentage of mothers ate vegetables and fruit once a week or more, whereas more fathers ate fast food weekly (Table 2).

**Table 2:** Traditional and imported food intake (time(s) per month) of ACCEPT follow-up population by gender

	Mothers (*N* = 101)				Fathers (*N* = 76)				*p*-value
	n (%)	Mean (SD)	Median (P25-P75)	Intake once a week or more n (%)	n (%)	Mean (SD)	Median (P25-P75)	Intake once a week or more n (%)	
**Traditional food**			13%^a^				15%^a^		
Marine mammals	76 (75%)	9.17 (10.47)	6.0 (3.0–9.0)	51 (67.1%)	50 (66%)	9.04 (10.83)	5.8 (2.5–10.0)	32 (64.0%)	0.826
Seabirds	89 (88%)	0.89 (1.20)	0.5 (0.0–1.0)	2 (2.2%)	64 (84%)	1.01 (0.88)	1.0 (0.5–1.5)	1 (1.6%)	0.112
Greenlandic fish	93 (92%)	6.28 (6.54)	4.5 (3.0–7.5)	49 (52.7%)	67 (88%)	5.77 (4.46)	4.5 (3.0–7.0)	35 (52.2%)	0.946
Shellfish	98 (97%)	2.60 (2.55)	2.0 (1.0–3.5)	15 (15.3%)	72 (95%)	3.45 (4.19)	2.5 (1.3–4.0)	17 (23.6%)	0.202
Dried fish	96 (95%)	4.25 (7.05)	2.5 (1.5–4.9)	27 (28.1%)	72 (95%)	3.63 (3.84)	2.5 (1.5–4.5)	20 (27.8%)	0.679
Terrestrial animals	94 (93%)	4.81 (5.23)	3.5 (2.0–5.5)	31 (33.0%)	66 (87%)	7.24 (10.35)	4.0 (2.5–7.0)	32 (48.5%)	0.191
Berries	95 (94%)	2.39 (4.26)	1.0 (0.5–2.5)	18 (18.9%)	68 (90%)	1.67 (2.73)	0.5 (0.5–1.8)	9 (13.2%)	0.094
**Imported food**			87%^a^				85%^a^		
Meat products	97 (96%)	19.18 (12.90)	17.0 (9.5–27.0)	95 (97.9%)	73 (96%)	17.92 (11.01)	14.5 (8.5–27.5)	70 (95.9%)	0.620
Carbohydrate foods	98 (97%)	37.09 (21.47)	39.0 (21.6–39.0)	95 (96.9%)	75 (99%)	35.70 (23.33)	30.3 (21.6–39.0)	74 (98.7%)	0.335
Sauce	100 (99%)	15.50 (10.71)	13.0 (4.3–30.4)	83 (83.0%)	76 (100%)	17.83 (12.72)	13.0 (4.3–30.4)	65 (88.2%)	0.312
Vegetables	101 (100%)	18.60 (12.39)	13.0 (13.0–30.4)	91 (90.1%)	76 (100%)	12.84 (11.45)	13.0 (4.3–13.0)	57 (75.0%)	0.001*
Fruits	99 (98%)	24.20 (18.06)	16.5 (13.0–33.4)	89 (89.9%)	73 (96%)	14.44 (15.1)	7.8 (4.3–17.8)	55 (75.3%)	< 0.001*
Fast food	98 (97%)	4.81 (5.47)	3.0 (1.5–6.0)	38 (38.8%)	73 (96%)	6.55 (6.76)	4.5 (2.5–7.5)	43 (58.9%)	0.005*
Sweets and Snacks	97 (96%)	34.56 (22.21)	32.5 (17.6–45.9)	95 (97.9%)	73 (96%)	32.78 (27.84)	22.9 (12.3–48.7)	72 (98.6%)	0.178

Differences between mothers and fathers were tested with a Mann Whitney U test. Bold p-values and * indicate significant difference (*p* ≤ 0.05). n (%): number of participants with information and percentages of the total number of participant in the group (N); SD: Standard Deviation; P25-P75: 25 percentile – 75 percentile; ^a^ The overall percentages of median intake of the main food groups, traditional (x) or imported food (y), were calculated by summing the medians of the main food groups and then the sum was divided by the total median intake (x + y). Also difference in the intake frequency > 1 per week were tested with chi-square test and difference were found for vegetables (*p* = 0.013), fruits (*p* = 0.013) and fast food (*p* = 0.013)

For mothers, the traditional and imported food was distributed as 13 and 87%, respectively. While for fathers, the percentages median food intake was 15% traditional and 85% imported food (Table 2).

#### Similarities and differences for parents living together

Within the study population, 73 mothers and fathers were living together. To assess the differences between the individual parent pairs, we subtracted the reported food intake of the father from the reported intake of the mother. There were no differences between parents in the intake frequency of the traditional food groups (Table 3). For the imported food groups, mothers had a significantly higher intake of fruit and vegetables and a less frequent intake of fast food than the fathers (Table 3).

**Table 3:** Parent differences and correlations between parents for traditional and imported food intake (time(s) per month)

	Parent difference (Mother - Father)				Unadjusted pairwise spearman correlation between parents			Adjusted pairwise spearman correlation between parents		
	n (%)	Mean (SD)	Median (P25-P75)	*p*-value	n	r_s_ (95%CI)	*p*-value	n	r_s_ (95%CI)	*p*-value
**Traditional foods**										
Marine mammals	40 (55%)	−0.14 (10.16)	0.0 (−4.0–1.5)	0.254	40 (55%)	0.51 (0.22; 0.72)	0.001*	38 (52%)	0.48 (0.17; 0.70)	0.005*
Seabirds	57 (78%)	− 0.23 (1.00)	0.0 (− 0.5–0.0)	0.061	57 (78%)	0.29 (0.02; 0.51)	0.031*	55 (75%)	0.26 (−0.01; 0.50)	0.062
Greenlandic fish	60 (82%)	0.11 (6.27)	0.0 (−2–1.25)	0.591	60 (82%)	0.48 (0.24; 0.66)	< 0.001*	57 (78%)	0.47 (0.22; 0.66)	< 0.001*
Shellfish	67 (92%)	−0.77 (5.13)	0.0 (−1.5–0.5)	0.179	67 (92%)	0.31 (0.07; 0.51)	0.011*	64 (88%)	0.30 (0.05; 0.51)	0.019*
Dried fish	65 (89%)	0.47 (4.80)	0.0 (− 1.0–1.0)	0.686	65 (89%)	0.43 (0.20; 0.62)	< 0.001*	62 (85%)	0.43 (0.19; 0.62)	0.001*
Terrestrial animals	60 (82%)	−2.44 (9.82)	0.0 (−1.5–1.0)	0.135	60 (82%)	0.53 (0.30; 0.70)	< 0.001*	57 (78%)	0.54 (0.31; 0.71)	< 0.001*
Berries	63 (86%)	1.07 (4.96)	0.0 (0.0–0.5)	0.052	63 (86%)	0.53 (0.31; 0.70)	< 0.001*	60 (82%)	0.50 (0.27; 0.68)	< 0.001*
**Imported food**										
Meat products	67 (92%)	0.69 (12.44)	0.0 (−4.6–6.9)	0.505	67 (92%)	0.37 (0.14; 0.57)	0.002*	64 (88%)	0.41 (0.17; 0.60)	0.001*
Carbohydrate foods	71 (97%)	−0.42 (22.00)	0.0 (−2.0–8.7)	0.678	71 (97%)	0.44 (0.22; 0.62)	< 0.001*	68 (93%)	0.44 (0.22; 0.62)	< 0.001*
Sauce	72 (99%)	−1.53 (13.00)	0.0 (−6.0–0.9)	0.240	72 (99%)	0.35 (0.12; 0.54)	0.003*	69 (95%)	0.29 (0.05; 0.50)	0.020*
Vegetables	73 (100%)	6.01 (14.69)	3.3 (0.0–17.4)	0.001*	73 (100%)	0.35 (0.12; 0.54)	0.003*	70 (96%)	0.29 (0.05; 0.49)	0.019*
Fruits	69 (95%)	10.51 (18.45)	10.2 (0.0–17.9)	< 0.001*	69 (95%)	0.36 (0.12; 0.55)	0.003*	66 (90%)	0.20 (−0.04; 0.43)	0.112
Fast food	68 (93%)	−2.53 (7.58)	−0.8 (−3.0–0.0)	< 0.001*	68 (93%)	0.42 (0.20; 0.61)	< 0.001*	65 (89%)	0.39 (0.15; 0.58)	0.002*
Sweets and Snacks	66 (90%)	0.64 (31.20)	0.4 (−11.4–14.5)	0.538	66 (90%)	0.28 (0.04; 0.49)	0.022*	63 (86%)	0.23 (−0.02; 0.45)	0.079

Number of parents living together and included in the analyses = 73. The parent differences were calculated by subtraction of the reported intake of the father from the reported intake of the mother, thus a difference of zero (0) reflect equal intake for the mother and father, negative values reflect higher intake for the father, and positive value reflect higher intake for the mother. The parent differences were tested with a Wilcoxon Signed Rank test, with median equals 0. Spearman correlations between parents intake (time(s) per months), are presented, raw and with adjustment for age differences, household income, and percentage for life in Greenland for both mother and father, highest education for both mother and father. Bold *p*-values and * indicate significant difference (*p* ≤ 0.05). n (%): number of participants with information and percentages of the total number of participant in the group (N); SD: Standard Deviation; P25-P75: 25 percentile – 75 percentile; r_s_ (95%CI): Spearman correlation coefficient with 95% confidence interval

Spearman pairwise correlations of the food groups between mothers and fathers were positive and moderate with an adjusted correlation coefficient ranging from 0.20 for fruits to 0.54 for terrestrial animals (Table 3). All correlations were significant in the unadjusted analyses, while seabirds, fruits, and sweets and snacks were non-significant in the adjusted analyses (Table 3).

#### Differences by geographical area (mothers and fathers pooled)

Table 4 shows the comparison of food intake by current living town. Significant lower consumption of terrestrial animals was observed for participants living in Nuuk compared to other towns. In comparison, significant higher intake frequency of fruit and vegetables were observed for participants living in Nuuk (Table 4). Nuuk and the other towns had different distributions regarding the percentage of median food intake for traditional and imported food, being 13 and 87% for participants in Nuuk, and 16 and 84% for participants living in other towns, respectively.

**Table 4:** Traditional and imported food intake (time(s) per month) of ACCEPT follow-up population by living place

	Nuuk (*N* = 119)			Other towns (*N* = 58)			*p*-value
	n (%)	Mean (SD)	Median (P25-P75)	n (%)	Mean (SD)	Median (P25-P75)	
**Traditional foods**			13%^a^			16%^a^	
Marine mammals	89 (75%)	8.05 (9.71)	5.5 (2.5–8.0)	37 (64%)	11.67 (12.18)	8.0 (4.0–16.0)	0.102
Seabirds	103 (87%)	0.93 (1.16)	0.5 (0.5–1.0)	50 (86%)	0.96 (0.90)	1.0 (0.0–1.5)	0.489
Greenlandic fish	108 (91%)	6.46 (6.32)	4.5 (3.5–7.8)	52 (90%)	5.26 (4.28)	4.0 (3.0–6.3)	0.269
Shellfish	114 (96%)	2.84 (2.64)	2.0 (1.0–3.5)	56 (97%)	3.22 (4.50)	2.0 (1.0–3.5)	0.615
Dried fish	113 (95%)	4.16 (6.61)	2.5 (1.5–4.5)	55 (95%)	3.62 (4.04)	2.5 (1.5–5.0)	0.870
Terrestrial animals	107 (90%)	4.73 (5.48)	3.5 (2.0–5.0)	53 (91%)	7.99 (10.90)	4.0 (2.5–7.5)	0.036*
Berries	111 (93%)	2.05 (3.14)	1.0 (0.5–2.5)	52 (90%)	2.19 (4.72)	1.0 (0.5–2.5)	0.935
**Imported food**			87%^a^			84%^a^	
Meat products	116 (98%)	18.48 (11.69)	17.0 (9.2–26.2)	54 (93%)	18.98 (13.08)	14.0 (8.3–29.0)	0.900
Carbohydrate foods	117 (98%)	34.92 (21.47)	35.4 (21.6–39.0)	56 (97%)	39.77 (23.62)	39.0 (27.8–46.1)	0.119
Sauce	118 (99%)	16.04 (11.48)	13.0 (4.3–30.4)	58 (100%)	17.46 (12.02)	13.0 (4.3–30.4)	0.529
Vegetables	119 (100%)	17.36 (11.89)	13.0 (13.0–30.4)	58 (100%)	13.59 (12.83)	13.0 (2.5–13.0)	0.013*
Fruits	118 (99%)	21.80 (17.12)	15.0 (5.8–32.4)	54 (93%)	16.25 (17.89)	13.0 (3.0–18.0)	0.011*
Fast food	117 (98%)	5.11 (4.54)	4.5 (2.0–6.3)	54 (93%)	6.51 (8.53)	3.5 (2.0–7.5)	0.973
Sweets and Snacks	115 (97%)	34.76 (22.87)	32.9 (15.0–50.9)	55 (95%)	31.80 (28.34)	24.0 (15.0–43.9)	0.153

Differences between Nuuk and other towns (including Sisimiut, Ilulissat, Kangerlussuaq and Qaqortoq) were tested with a Mann Whitney U test. Bold *p*-values and * indicate significant difference (*p* ≤ 0.05). n (%): number of participants with information and percentages of the total number of participant in the group (N); SD: Standard Deviation; P25-P75: 25 percentile – 75 percentile; ^a^The overall percentages of median intake of the main food groups, traditional (x) or imported food (y), were calculated by summing the medians of the main food groups and then the sum was divided by the total median intake (x + y)

When comparing food intake frequency by the region where the participants had lived the longest, only the frequency of marine mammal intake was statistically significant different among regions (*p* = 0.027, not shown). Marine mammals were most frequently consumed in Disko bay (8.5 times/month, *n* = 25), followed by other Greenlandic regions (North, South, East) (8.0 times/month, *n* = 7), and West (5.3 times/month, *n* = 82), and less frequent by those who have lived > 50% of their life in other countries (1.8 times/month, *n* = 4). None of the other food groups differed by region (not shown).

#### Age differences (mothers and fathers pooled)

Table 5 shows the comparison of the median higher and lower age groups. The older age group (≥34.3 years) reported a more frequent intake of Greenlandic fish and fruit and less frequent intake of meat products and fast food compared to the younger age group (< 34.3 years) (Table 5). The two age groups had similar distributions regarding the percentage of median food intake for traditional and imported food, with 13 and 87% for the younger group and 14 and 86% for the older group (Table 5).

**Table 5:** Traditional and imported food intake (time(s) per month) of the ACCEPT follow-up population by age

	< 34.3 years (*N* = 88)			≥34.3 years (*N* = 89)			*p*-value
	n (%)	Mean (SD)	Median (P25-P75)	n (%)	Mean (SD)	Median (P25-P75)	
**Traditional foods**			13%^a^			14%^a^	
Marine mammals	67 (76%)	9.23 (11.93)	6.0 (2.5–9.0)	59 (66%)	8.98 (8.89)	6.0 (3.0–11.0)	0.644
Seabirds	77 (88%)	0.89 (0.99)	0.5 (0.0–1.0)	76 (85%)	0.99 (1.17)	1.0 (0.5–1.5)	0.284
Greenlandic fish	78 (89%)	5.40 (5.47)	4.0 (2.5–6.0)	82 (92%)	6.70 (5.97)	5.0 (3.5–8.5)	0.012*
Shellfish	85 (97%)	2.59 (2.46)	2.0 (1.0–3.5)	85 (96%)	3.33 (4.04)	2.0 (1.5–4.0)	0.201
Dried fish	83 (94%)	3.54 (4.09)	2.5 (1.5–4.5)	85 (96%)	4.41 (7.22)	2.5 (1.5–5.0)	0.348
Terrestrial animals	79 (90%)	5.43 (6.97)	3.5 (2.0–5.8)	81 (91%)	6.19 (8.61)	3.5 (2.5–6.0)	0.544
Berries	81 (92%)	2.44 (4.57)	1.0 (0.5–2.5)	82 (92%)	1.75 (2.55)	1.0 (0.5–2.5)	0.876
**Imported food**			87%^a^			86%^a^	
Meat products	83 (94%)	19.70 (9.50)	20.3 (10.3–27.5)	87 (98%)	17.63 (14.14)	13.4 (8.5–24.1)	0.032*
Carbohydrate foods	86 (98%)	35.90 (19.41)	39.0 (21.6–39.0)	87 (98%)	37.07 (24.82)	39.0 (21.6–39.0)	0.958
Sauce	87 (99%)	16.30 (11.10)	13.0 (4.3–30.4)	89 (100%)	16.71 (12.22)	13.0 (4.3–30.4)	0.963
Vegetables	88 (100%)	15.18 (12.29)	13.0 (4.3–30.4)	89 (100%)	17.06 (12.31)	13.0 (13.0–30.4)	0.227
Fruits	86 (98%)	17.51 (17.13)	14.0 (4.3–28.5)	86 (97%)	22.61 (17.60)	16.0 (7.5–33.4)	0.013*
Fast food	84 (96%)	6.35 (6.86)	4.5 (2.5–7.2)	87 (98%)	4.79 (5.18)	3.0 (1.5–6.0)	0.031*
Sweets and Snacks	85 (97%)	35.34 (26.20)	26.1 (15.0–52.6)	85 (96%)	32.26 (23.21)	29.0 (15.4–43.2)	0.578

Differences between age groups were tested with a Mann Whitney U test. Bold p-values and * indicate significant difference (p ≤ 0.05). n (%): number of participants with information and percentages of the total number of participant in the group (N); SD: Standard Deviation; P25-P75: 25 percentile – 75 percentile; ^a^The overall percentages of median intake of the main food groups, traditional (x) or imported food (y), were calculated by summing the medians of the main food groups and then the sum was divided by the total median intake (x + y)

The age comparison stratified by gender showed similar tendencies as pooled data; however, only few of the results were significant (Additional file 3). Among mothers, Greenlandic fish intake was significantly lower in the younger age group (< 34.0y ears, 4.0 times/month) than in the older age group (≥34.0 years, 6.0 times/months) (*p* = 0.010) (Additional file 3: Table S3A). Among fathers, fruit was significantly lower in the younger age group (< 36.7 years, 5.8 times/month) than in the older age group (≥36.7 years, 14.3 times/months) (*p* = 0.015) (Additional file 3: Table S3B).

#### Correlations with socioeconomic and lifestyle factors (mothers and fathers pooled)

**Fig. 3:**
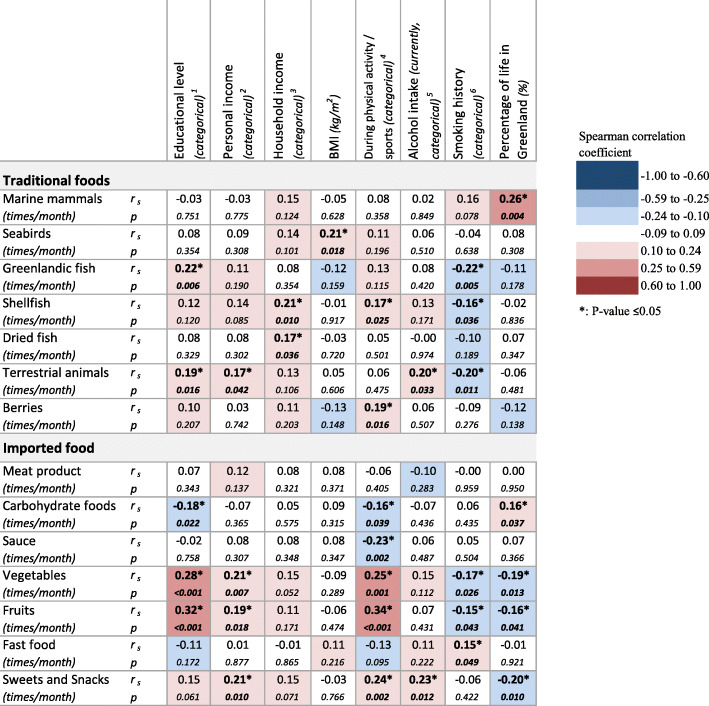
Spearman correlations between frequency intake of food groups and socioeconomic and lifestyle factors. Spearman correlation coefficients (r_s_ in the upper line) with p-value for the correlation (below in smaller text size). Blue colors indicate negative correlations and red colors positive correlations, bold text indicate a significant correlation. Some variables were categorical with the following categories; ^1^Educational level (Primary School, High School, Technical college, and University), ^2^Personal income/ ^3^Household Income (< 100.000, 100.000–250.000, and > 250.000 DKK/year), ^4^Performing physical activities/sports (No and Yes), ^5^Current alcohol intake (0 drinks/week, 1–7 drinks/week, and ≥ 8 drinks/week), ^6^Smoking history (Never, Former, and Current). For Personal income, Household Income, and Alcohol intake the answer possibility “Don’t know” was omitted in the analysis

Several socioeconomic and lifestyle factors were significantly correlated with intake frequency of both traditional and imported food groups, and the correlations were weak to moderate (Fig. 3).

As shown in Fig. 3, socioeconomic factors (education and income) were generally positively correlated with the intake frequency of the food groups. High educational level was significantly positively correlated with frequent intake of Greenlandic fish, terrestrial animals, vegetables, and fruits, but negatively with carbohydrate foods. Significant positive correlations were also seen between high personal income and intake of terrestrial animals, vegetables, fruit, and sweets and snacks and between high household income and intake of shellfish and dried fish (Fig. 3).

For the lifestyle factors, BMI was positively correlated with seabird intake. Physical activity/sports was significantly positive correlated with intake of shellfish, berries, vegetables, fruit and sweets and snacks, and negatively with carbohydrate foods and sauce intake. High alcohol intake was correlated with terrestrial animals and sweets and snacks intake. Smoking history was correlated with several food groups. Current smokers had a less frequent intake of Greenlandic fish, shellfish, terrestrial animals, vegetables, and fruits than never smokers, but a more frequent fast food intake. Furthermore, the percentage of life in Greenland was significantly positive correlated with intake of marine mammals and carbohydrate foods and negatively with vegetables, fruit, and sweets and snacks (Fig. 3).

#### Changes from inclusion at pregnancy to the present follow-up (mothers only)

To assess changes from inclusion at pregnancy to follow-up for the mothers, the individual differences were calculated by subtraction intake at inclusion from intake at follow-up. The mothers generally reported similar intake at inclusion at pregnancy and follow-up (after 3–5 years) (Table 6). However, at follow-up, they reported significantly less frequent intake of seabirds and fruits and more frequent imported meat intake compared to inclusion at pregnancy (Table 6).

**Table 6:** Changes and correlations for food intake for mothers between inclusion and follow-up (3–5 years)

	Individual difference (Follow-up – Inclusion)				Unadjusted pairwise spearman correlation			Adjusted pairwise spearman correlation		
	n (%)	Mean (SD)	Median (P25-P75)	*p*-value	n	r_s_ (95%CI)	*p*-value	n	r_s_ (95%CI)	*p*-value
**Traditional foods**										
Marine mammals	68 (67%)	1.02 (11.0)	0.0 (−2.8–2.3)	0.800	68 (67%)	0.33 (0.10; 0.53)	0.006*	33 (32%)	0.31 (0.04; 0.59)	0.047*
Seabirds	89 (87%)	−0.49 (2.0)	0.0 (−1.0–0.0)	0.001*	89 (87%)	0.37 (0.17; 0.53)	< 0.001*	44 (43%)	0.43 (0.15; 0.64)	0.003*
Greenlandic fish	92 (90%)	−0.13 (7.2)	0.0 (−3.1–1.5)	0.368	92 (90%)	0.40 (0.21; 0.56)	< 0.001*	43 (42%)	0.33 (0.03; 0.57)	0.023*
Shellfish	96 (94%)	−0.02 (2.5)	0.0 (−1.3–1.0)	0.492	96 (94%)	0.50 (0.33; 0.64)	< 0.001*	46 (45%)	0.53 (0.28; 0.71)	< 0.001*
Dried fish	95 (93%)	−0.70 (11.6)	0.0 (−2.0–1.0)	0.386	95 (93%)	0.37 (0.18; 0.53)	< 0.001*	47 (46%)	0.39 (0.12; 0.61)	0.006*
Terrestrial animals	94 (92%)	−0.31 (5.2)	−0.4 (−1.5–1.0)	0.211	94 (92%)	0.52 (0.36; 0.65)	< 0.001*	46 (45%)	0.52 (0.26; 0.70)	< 0.001*
Berries	92 (90%)	0.90 (4.7)	0.0 (−0.5–1.0)	0.139	92 (90%)	0.27 (0.06; 0.45)	0.011*	45 (44%)	0.22 (−0.08; 0.48)	0.137
**Imported food**										
Meat products	95 (93%)	4.23 (17.3)	2.5 (−3.6–13.0)	0.011*	95 (93%)	0.06 (−0.15; 0.25)	0.598	46 (45%)	0.04 (−0.26; 0.32)	0.813
Carbohydrate foods	98 (96%)	−2.56 (28.6)	0.0 (−17.4–10.5)	0.343	98 (96%)	0.24 (0.04; 0.42)	0.017*	49 (48%)	0.26 (−0.03; 0.50)	0.078
Sauce	101 (99%)	−0.57 (11.3)	0.0 (−8.7–1.8)	0.676	101 (99%)	0.46 (0.29; 0.60)	< 0.001*	49 (48%)	0.46 (0.21; 0.66)	0.001*
Vegetables	102 (100%)	0.34 (13.8)	0.0 (0.0–10.5)	0.812	102 (100%)	0.35 (0.17; 0.51)	< 0.001*	50 (49%)	0.39 (0.13; 0.66)	0.006*
Fruits	99 (97%)	−4.82 (18.9)	−1.5 (−17.4–4.0)	0.007*	99 (97%)	0.43 (0.26; 0.58)	< 0.001*	47 (46%)	0.46 (0.20; 0.66)	0.001*
Fast food	97 (95%)	−0.29 (6.9)	0.0 (−2.0–1.5)	0.486	97 (95%)	0.38 (0.20; 0.54)	< 0.001*	47 (46%)	0.43 (0.16; 0.64)	0.002*
Sweets and Snacks	94 (92%)	0.89 (26.6)	2.5 (−15.6–15.6)	0.536	94 (92%)	0.36 (0.17; 0.53)	< 0.001*	46 (45%)	0.35 (0.07; 0.58)	0.014*

The analyses were conducted on mothers only. The individual differences were calculated by subtraction of the reported intake from inclusion from the reported intake at follow-up, thus a differences of zero (0) reflect equal intake at inclusion and follow-up, negative values reflect higher intake at inclusion, and positive value reflect higher intake at follow-up. Mean (SD) and median (P25-P75) difference in food intake is given as time(s)/month. The individual differences were tested with a Wilcoxon Signed Rank test, with median equals 0. Bold p-value and * indicate significant difference (*p* < 0.05). Spearman correlations between intake of traditional and imported food groups (time(s) per months) at inclusion and follow-up (3–5 years). Adjusted correlations is adjusted for time from inclusion to follow-up, change in education level, change in household income, change in BMI, and change in parity. n (%): number of participants with information and percentages of the total number of participant in the group (N); SD: Standard Deviation; P25-P75: 25 percentile – 75 percentile; r_s_ (95%CI): Spearman correlation coefficient with 95% confidence interval

Spearman pairwise correlations for the food groups from inclusion at pregnancy and this follow-up study were all positive (Table 6). Most of the correlations were moderate, and the adjusted correlation coefficient ranged from 0.22 for berries to 0.53 for shellfish, except for meat products, which was weaker (*r*_*s*_ = 0.035). All correlations were significant in the unadjusted analyses, except for meat products, while berries and carbohydrate foods were non-significant in the adjusted analyses. The correlation estimates were similar in the unadjusted and adjusted analyses (Table 6).

## Discussion

In the present study, we followed up on the food intake of women included in the ACCEPT birth cohort and children’s fathers. The distribution between the intake frequency for traditional and imported food groups was 14 and 86%, respectively. Intake frequency for several of the food groups differed by gender (vegetables, fruit, and fast food), living town (terrestrial animals, vegetables, and fruits), and age (Greenlandic fish, meat products, fruits, and fast food). Socioeconomic and lifestyle factors significantly correlated with the intake frequency of both traditional and imported food groups. Smoking negatively correlated with intake of Greenlandic fish, shellfish, terrestrial animals, vegetables, and fruit and positively correlated with fast food intake. Generally, there were significant correlations in the food groups’ intake between the parents living together; however, the mothers reported a more frequent intake of vegetables and fruit and less for fast food. Few changes in the mother’s dietary habits from inclusion (at pregnancy) to this follow-up study (3–5 years later) were found, with less frequent intake of seabirds and fruit and more frequent meat intake.

In general, the proportion of traditional and imported food and the intake frequencies of the different food groups was similar to previously reported for the ACCEPT cohort [18, 19]. Compared to other studies in Greenland, more participants reported eating fish a least once a week in the present study (52.5%) than in the latest Population Health Survey from 2018 (42.8%) [17]. However, the percentage in the present study was comparable with two older Population Health Surveys from 2005 to 2010 (56.0%) and 2014 (50.2%) [16, 24]. The percentage reporting a daily fruit intake in the Population Health Surveys (2005–2010: 35.9%, 2014: 44.9%, and 2018: 35.9%) [16, 17, 24] are higher than the present study, where 30.8% consume fruit daily, with a difference between men (16.4%) and women (41.4%) (data not shown).

The health effects of the changing diet in Greenland is not fully understood. However, increased incidence in overweight and obesity in Greenland, may be linked to the western lifestyle and diet transition. Neither a diet consisting totally of traditional or imported food may be optimal for health in Greenland. The transition has resulted in increased consumption of sugar and saturated fat, and decreasing intake of n-3 fatty acids [5]. On the positive side, the intake of dietary fibre increased and the reduced intake of marine mammals and seabirds have resulted in reduced POP exposure. However, it must be noted that the POP exposure can be significantly reduced by omitting a few traditional food items (such as liver, kidney and blubber from seal and whales), without changing the intake of selenium and n-3 fatty acids significantly [25, 26].

### Gender and parent differences in food intake

The intake frequency of traditional food groups was similar for mothers and fathers (Table 2); however, the percentage of traditional food was higher among the fathers (15%) compared to the mothers (13%). A similar difference between men and women has previously been seen in Greenland [24], as well as among other Indigenous populations, including the Sami population in Northern Norway [27] and the Eeyou Istchee communities in northern Quebec [28].

Mothers had significantly more frequent intake of fruit and vegetables and less frequent fast food intake (Table 2). These results are in line with previous observations, both in Greenland and internationally. Generally, studies find that women have a healthier lifestyle and diet [29, 30]. Among the Sami population in Northern Norway, fruit and vegetable intake positively correlated with the female gender [27]. In Greenland, women were more likely to comply with the national dietary guidelines [17], and the mean energy intake from fast food was higher for men (1850 kJ/day) than for women (1485 kJ/day) [24].

Among parents living together, intake from the food groups, both traditional and imported, was positively correlated (Table 3). However, mothers had a more frequent intake of vegetables and fruit and less frequent intake of fast food than the fathers, similarly to what was seen in the gender analyses (Table 2). The positive correlations are not surprising, as the parents share household larders and mostly consume main meals together. Other studies have found that couples tend to eat similar foods and have similar nutrient intakes [31–37]; however, female partners were often restricted in dinner choices by their male partners’ preferences for meat, fewer vegetables, and little food variety [38]. In the present study, the mothers reported a more healthy diet with more frequent intake of fruit and vegetables and less fast food intake than the fathers. The result may indicate that even though the parents most often eat together, the mothers more often eat fruit and vegetables as side dishes, whereas the fathers more often visit fast food places, for instance, at lunchtime.

### Food differences by geographical area

The living place can affect the hunting possibility and the availability of traditional foods, and the degree of urbanization also influences the availability and supply of imported food. In the present study, terrestrial animal intake was lower in Nuuk than in the other towns (Sisimiut and Ilulissat), while vegetables and fruit were more frequent consumed in Nuuk. The percentage of traditional food was also higher among participants from the other towns (16%) than Nuuk (13%). The availability of the food items may very well explain these differences. Previously, the Population Health Surveys have reported that participants living in settlements were more likely to eat a meal from their own hunt/catch once a week (80%) than participants from towns (39%) and the main city Nuuk (20%) [24]. In compliance with the present study, in the study on Health Behavior in School-aged Children, the proportion of children eating fruit and vegetables daily was higher in Nuuk than in other towns and settlements [39].

In the ACCEPT cohort, geographical differences were investigated based on the region where the women had lived the longest and not by the current living place [18, 19]. Differences among regions were seen for terrestrial animals, berries, sauce, and fast food. Data from 2010 to 2011 showed a less frequent intake of terrestrial animals and berries in Disko Bay than in West, North, South, and East [18], but this difference was not seen during 2013–2015 [19] and neither in the present study. However, for terrestrial animals, we found that the participants living in Ilulissat (Disko Bay) did have a non-significantly less frequent intake (3.0 times/month) than participants living in Sisimiut (West, 5.0 times/month) and Nuuk (West, 3.5 times/month) (data not shown).

### Age differences in food intake

The older age group (≥34.3 years) had a healthier lifestyle than the younger group (< 34.3 years), with more frequent fish and fruit intake and less frequent fast food intake. Similar results have been shown in other studies, internationally [40] and in Greenland [17–19, 24]. For the young population (18–24 years) in Greenland, the energy consumption from traditional food is lower, the intake of sugar and fast food is higher, and they are less likely to comply with the national dietary guidelines than the older population of ≥60 years of age [17, 24]. Similar to the results in the present follow-up study, at inclusion into the ACCEPT cohort, the younger pregnant women did also have a more frequent fast food intake than the older pregnant women [18, 19]. Thus, the present study is in accordance and confirms previous studies in Greenland.

### Correlations with socioeconomic and lifestyle factors

Participants with high socioeconomic status seem to have a healthier lifestyle than participants with low socioeconomic status. High socioeconomic status (high educational level and high income) positively correlated with more frequent intake of Greenlandic fish, shellfish, dried fish, terrestrial animals, vegetables, and fruit. However, high personal income was also correlated with more frequent intake of sweets and snacks. Other studies have also shown that high educational status tends to correlate with consumption of a better-quality diet [41, 42]. In Greenland, occupation was previously associated with food insecurity (experienced times without any food or money to buy within the last 12 months) among adults, with more unemployed having experienced food insecurity (33%) compared to hunters/fishers (12%) and skilled/trained workers (4%) [16].

We also found that lifestyle factors (BMI, physical/sport activity, alcohol intake, and smoking history) correlated with the intake of several food groups. Only intake of seabird was significantly correlated with BMI. Physical/sport activities correlated positively with shellfish, berries, vegetables, fruit and sweets and snacks but negatively with carbohydrate food and sauce. Furthermore, alcohol intake correlated positively with the intake of terrestrial animals and sweets and snacks. Current smoking was negatively correlated with intake of Greenlandic fish, shellfish, terrestrial animals, vegetables, and fruit, but positively with fast food. Cigarette smoking is a significant source of oxidative stress. One potential mechanism for its problematic health effects is that smoking contributes to endogenous oxidant formation through the inflammatory-immune response [43, 44]. Thus, it is worrying that current smoking is negatively associated with vegetable and fruit intake, as antioxidants consumed in the diet may act to mitigate against some of the adverse effects of cigarette smoking [45]. Other studies have shown that smokers have lower blood levels of antioxidants (ascorbic acid, α-carotene, β-carotene, vitamin A and E) than non-smokers [45], which might be due to both the oxidants in cigarette smoke and lower intake of antioxidants in smokers than non-smokers [45]. Smoking continues to be a public health problem in Greenland with more than half of the general population being current smokers [17] and 29–46% of the pregnant women in the ACCEPT cohort (2010–2015) smoked during pregnancy [18, 19]. A connection of smoking with an unhealthy diet may worsen the health problems further.

### Changes from inclusion at pregnancy to this follow-up study (3–5 years later)

Overall, the food intake frequency of both the traditional and imported food groups in the present follow-up study was similar to previously reported frequencies in the ACCEPT birth cohort [18, 19].

However, the individual intake frequency of fruit has decreased with a median of 1.5 times/months from pregnancy to this follow-up study, and meat intake increased a median of 2.5 times/month. The decreased frequency in fruit intake may be due to several factors. It may be explained by a decreased fruit intake in the general population seen in the same period in the Population Health Surveys [16, 17], or the women may have been more aware of having a healthy lifestyle during their pregnancy. A Canadian study investigated dietary changes during pregnancy and found that pregnant women reported increasing intake of milk products, fruit, and sweet items and decreased or eliminated intake of caffeine, alcohol, and meats during pregnancy [46]. Increased fruit intake during pregnancy has also been reported in several other studies (reviewed by SE Hillier and EK Olander [47]). In line with the increase in meat intake in the present follow-up study from inclusion at pregnancy, the Canadian study found decreased or eliminated meat intake during pregnancy, which were mainly due to concern for the baby’s health, aversion, and nausea [46].

It is, to our knowledge, the first time that longitudinal dietary changes are reported in Greenland. The results may suggest that the women changed their diet during pregnancy and that the dietary habits have “normalized” to non-pregnancy habits after pregnancy. However, we do not have any measurements of the women’s intake before pregnancy, and it is possible that the women did not change diet during pregnancy but have changed the diet afterward.

### Strengths and limitations of the study

To estimate the food intake of the different food groups, participants were asked to fill out the FFQ with 65 traditional and imported food items based on their intake during the last 12 months. FFQ is a widely used dietary assessment method, especially in epidemiological studies, and compared to other dietary assessment methods, it is relatively simple, cost-effective, and time-efficient. The self-reported food intake may be subject to recall bias, as the participants must remember their intake 12 months back in time, which can be difficult. The portion size was not included in the questionnaire, and it is possible that the individual amount consumed differs, even though we do not see any difference in the frequency. However, other studies have reported that the between-person variation in portion size was smaller and less important than the variation in frequency [48]. A previous study in Greenland found good agreement between a very similar FFQ and duplicate portions, even though there was a slight overestimation of traditional food and underestimation of sweets in the FFQ [4]. Due to lack of nutritional data for some food items and missing portion sizes and seasonal information, we have not been able to calculate the nutritional intake for the participants and compare with Nutrition Recommendations.

For some of the Greenlandic food items, intake may depend on hunting season and availability; thus, we tried to include the seasonal variation in the FFQ. However, season information was missing for 5–56% in the Greenlandic food items, and we were unable to include the information in the analyses. However, looking at the available seasonal information (Additional file 2: Table S2B), the season variation was limited for most items, but for some items, the differences between the percentage of eating the food item in spring/summer and autumn/winter were more than 15%. For seabirds (guillemot, common eider, and kittiwake), hare and grouse more participants reported intake in the autumn/winter than in spring/summer, while some marine mammals (minke whale, fin whale, porpoise/grind and hooded seal), champs, and some fish (capelin and trout) were eaten by more participants in the spring/summer than in the autumn/winter.

Due to limited funding and the logistic difficulties connected with data collection in the remote settlements in Greenland, we could only follow up on the families in the three biggest towns (Nuuk, Sisimiut, and Ilulissat). However, Greenlandic statistic reports show that 50% of the Greenlandic population live in these three towns, with a distribution (64% living in Nuuk, 19% in Sisimiut, and 16% in Ilulissat) comparable with the distribution in the present study (67% in Nuuk, 21% in Sisimiut, and 11% in Ilulissat) [49]. Thus, even though the present study results may not reflect the food intake for the total Greenlandic population, the study population represents at least half of the Greenlandic population with a distribution reflecting the population among the included towns.

In the general Greenlandic population, a more western lifestyle and higher socioeconomic status are generally seen in the towns, especially in Nuuk [17]. Due to the fact that only ACCEPT women in Ilulissat, Sisimiut and Nuuk were followed up in this study, they differed slightly from the ACCEPT birth cohort women (2013–2015) not included in this ACCEPT follow-up study; the women included in this follow-up study were older at delivery (median: 29.2 versus 27.4, *p* = 0.001), less likely to smoke during pregnancy (20.6% versus 38.6%, p = 0.001), had higher educational level (66.3% with technical college or university degree versus 42.3%, *p* < 0.001) and had higher marine food intake as indicated by the n-3/n-6 fatty acids ratio (median: 0.24 versus 0.21, p = 0.001) (data not shown). The women included in this study were similar to the non-included ACCEPT women with regard to BMI, parity, and alcohol consumption (before and during pregnancy) at inclusion (data not shown).

## Conclusion

In the present study, we follow-up on 101 women included in the ACCEPT birth cohort (2013–2015) to assess the current dietary intake for the mother and the children’s fathers using a FFQ including traditional Greenlandic food items and imported food items. Comparable to previously data in the ACCEPT cohort [18, 19] and other Greenlandic studies [17], the distribution between the intake frequency for traditional and imported food groups was 14 and 86%, respectively. In line with other studies, mothers seem to have more healthy dietary habits, although significant positive correlations were seen between parents living together. Age, living town, and several socioeconomic and lifestyle factors were related to the food frequency intake. This indicates that social classes highly influence the dietary habits, participants with higher education generally have healthier dietary habits, while current smokers generally eat more unhealthy food than never smokers.

The results from the present follow-up study suggest that the women changed their diet during pregnancy and that the dietary habits seems to go back to non-pregnancy habits after pregnancy. This suggest that some of the women adapted to the food recommendations given to pregnant women in Greenland. However, a study with dietary information before, during and after pregnancy is needed to confirm this.

For further diet recommendations, it is important to monitor the dietary and lifestyle habits frequently to follow the transition in Greenland. The health implications of the ongoing dietary transition in Greenland are complex, as both traditional and imported food can positively and negatively affect health, and the cultural aspect of traditional food intake must as well be taken into consideration. However, by omitting a few traditional food items (from seal and whale), the beneficial nutritional factors from the traditional diet can be kept while the intake of contaminants are reduced.

## Supplementary Information

**Additional file 1.**

**Additional file 2.**

**Additional file 3.**

## Data Availability

The datasets generated and/or analysed during the current study are not publicly available due to identifiable content, but are available from the corresponding author on reasonable request and ethical and legal approval.

## Abbreviations

ACCEPT: Adaptation to Climate Change, Environmental Pollution, and dietary Transition
BMI: body mass index
FFQ: food frequency questionnaire
n-3: Omega 3 fatty acids
POPs: persistent organic pollutants
