# Supplementary material for: Dietary habits among men and women in West Greenland: follow-up on the ACCEPT birth cohort
Source: BMC Public Health. 2021 Jul 19;21:1426. doi: 10.1186/s12889-021-11359-7 (PMC8290613; doi:10.1186/s12889-021-11359-7)
Supplement: Supplementary file 1 — Additional file 1. [file 12889_2021_11359_MOESM1_ESM.pdf]

Additional file 1

Dietary habits among men and women in West Greenland: follow-up on the ACCEPT birth cohort

Main food groups and calculations of food intake

The food items were divided into seven main traditional food groups: Marine mammals, Seabirds, Fish, Dried fish, Shellfish, Terrestrial animals, and Berries and seven main imported food groups: Meat products, Carbohydrate foods, Sauce, Fruit, Vegetables, Fast food, and Sweets and Snacks. Table S1A show the specific food items categorized in each main food group are show.

Table S1A: Food items included in the main food groups

Main food groups	Food items included
Traditional food groups	
Marine mammals	Seal (Hooded seal, and other seals), Whale (Minke whale, Fin whale, Beluga whale, Narwhale, and Harbor porpoise/Grind), Dried whale and seal meat (Narwhale or Beluga whale, Fin- and Minke whale, and Seal), Blubber (Seal blubber, Fin- and Minke whale blubber, and Narwhale blubber), Suaasat with seal or whale, Walrus, and Polar bear
Seabirds	Guillemot, Common eider, Black-legged Kittiwake, and Other seabirds
Greenlandic fish	Trout, Cod, Greenlandic Halibut, Atlantic Halibut, Redfish, Atlantic Salmon, Atlantic Wolfish, and Capelin
Shellfish	Shrimps, Clamps, and Crab
Dried fish	Dried Greenlandic Halibut, Dried Capelin, Dried Cod, Dried Wolfish, and Other dried fish
Terrestrial animals	Grouse, Greenlandic lamb or sheep, Caribou (wild-living Arctic reindeer), Muskox, and Hare
Berries	All berries
Imported food groups	
Meat products	Chicken or turkey, Pork, Beef, Imported lamb, and Imported fish
Carbohydrate foods	Potato, Pasta, and Rice
Sauce	All sauces
Vegetables	All vegetables
Fruits	Fresh fruit, Canned fruit, and Frozen fruit
Fast food	Pizza, Ready-made meals, and Take away from grill or café
Sweets and Snacks	Milk chocolate, Dark chocolate, Candy, Chips, Dried Fruit, Nuts, and Cake

For each food item, a frequency score was applied transforming all categories from 'never' to 'several times a day' into number of times consumed per month (Table S1B). The intake of each main food group was calculated by summing frequency score of included food items for each participant (example see Table S1C).

Table S1B: Consumption categories transformed into frequency score

Consumption categories from the FFQ	Frequency score (time(s) per month)
"Never"	0.0
"Less than once a month"	0.5
"1 time a month"	1.0
"2-3 times a month"	2.5
"1 time a week"	4.3
"2-4 times a week"	13.0
"Every day/Almost every day"	30.4
"Several times a day"	45.6

Table S1C: Example of calculation of food intake a month for the main food group "Shellfish" for four random participants

Participant	Shrimps		Clams		Crabs		Sum Shellfish
	Answer in FFQ	*Score (times/months)*	*Answer in FFQ*	*Score (times/months)*	*Answer in FFQ*	*Score (times/months)*	*Sum of score for shrimps, clams, and crabs*
1	1 time a week	= 4.3 times/month	2-3 times a month	= 2.5 times/month	2-3 times a month	= 2.5 times/month	9.3 times a month
2	1 time a month	= 1.0 times/month	Less than once a month	= 0.5 times/month	Less than once a month	= 0.5 times/month	2 times a month
3	Less than once a month	= 0.5 times/month	Never	= 0.0 times/month	Never	= 0.0 times/month	0.5 times a month
4	2-4 times a week	= 13.0 times/month	1 time a month	= 1.0 times/month	Less than once a month	= 0.5 times/month	14.5 times a month
