# Supplementary material for: Dietary habits among men and women in West Greenland: follow-up on the ACCEPT birth cohort
Source: BMC Public Health. 2021 Jul 19;21:1426. doi: 10.1186/s12889-021-11359-7 (PMC8290613; doi:10.1186/s12889-021-11359-7)
Supplement: Supplementary file 2 — Additional file 2. [file 12889_2021_11359_MOESM2_ESM.pdf]

Additional file 2

Dietary habits among men and women in West Greenland: follow-up on the ACCEPT birth cohort

Intake of food groups and food items for mothers and fathers

Table S2A: Traditional and imported food intake (time(s) a month) for mothers and fathers (N=177)

	n (%)	Mean (SD)	Median (P25-P75)	Min-Max	Intake once a week or more n (%)
**Traditional food**			14 %[a]		
Marine mammals	126 (71%)	9.12 (10.57)	6.0 (3.0-9.5)	0.0-54.4	83 (65.9%)
Seabirds	153 (86%)	0.94 (1.08)	1.0 (0.0-1.5)	0.0-9.1	3 (2.0%)
Greenlandic fish	160 (90%)	6.07 (5.75)	4.5 (3.0-7.5)	0.0-45.5	84 (52.5%)
Shellfish	170 (96%)	2.96 (3.36)	2.0 (1.0-3.5)	0.0-30.4	32 (18.8%)
Dried fish	168 (95%)	3.98 (5.88)	2.5 (1.5-4.5)	0.0-62.8	47 (28.0%)
Terrestrial animals	160 (90%)	5.81 (7.83)	3.5 (2.0-6.0)	0.0-61.3	63 (39.4%)
Berries	163 (92%)	2.09 (3.70)	1.0 (0.5-2.5)	0.0-30.4	27 (16.6%)
**Imported food**			86 %[a]		
Meat products	170 (96%)	18.64 (12.11)	15.3 (8.8-27.0)	2.0-92.2	165 (97.1%)
Carbohydrate foods	173 (98%)	36.49 (22.24)	39.0 (21.6-39.0)	0.0-136.8	169 (97.7%)
Sauce	176 (99%)	16.51 (11.64)	13.0 (4.3-30.4)	0.0-45.6	148 (84.1%)
Vegetables	177 (100%)	16.13 (12.30)	13.0 (4.3-30.4)	0.0-45.6	148 (83.6%)
Fruits	172 (97%)	20.06 (17.50)	14.5 (5.3-31.9)	0.0-78.5	144 (83.7%)
Fast food	171 (97%)	5.55 (6.10)	4.0 (2.0-6.3)	0.0-47.1	81 (47.4%)
Sweets and Snacks	170 (96%)	33.80 (24.73)	28.5 (15.0-46.8)	1.5-153.5	167 (98.2%)

n (%): number of participants with information and percentages of the total number of participant (N); SD: Standard Deviation, P25-P75: 25 percentile – 75 percentile; [a] The overall percentages of median intake of the main food groups, traditional (x) or imported food (y), were calculated by summing the medians of the main food groups and then the sum was divided by the total median intake (x+y).

Table S2B: Intake of traditional and imported food items (time(s) a month) and seasonal information for mothers and fathers together (N=177)

	n (%)	Mean (SD)	Median (P25-P75)	Intake once a week or more n (%)	Spring/ Summer[a]	Autumn/ Winter[a]	Missing information on season[b]
**Traditional food items**							
Hooded seal	154 (87%)	0.27 (0.69)	0.0 (0.0-0.5)	3 (1.9%)	16 (84.2%)	9 (47.4%)	49 (28%)
Other seal	163 (92%)	1.30 (2.33)	0.5 (0.5-1.0)	13 (8.0%)	51 (85.0%)	51 (85.0%)	88 (50%)
Minke whale	159 (90%)	0.52 (0.62)	0.5 (0.0-0.5)	1 (0.6%)	45 (91.8%)	37 (75.5%)	76 (43%)
Fin whale	162 (92%)	0.37 (0.64)	0.0 (0.0-0.5)	2 (1.2%)	28 (84.8%)	22 (66.7%)	59 (33%)

Beluga whale	167 (94%)	0.64 (1.23)	0.5 (0.0-0.5)	2 (1.2%)	37 (74.0%)	45 (90.0%)	65 (37%)
Narwhale	169 (96%)	0.53 (0.68)	0.5 (0.0-0.5)	1 (0.6%)	37 (75.5%)	47 (95.9%)	70 (40%)
Porpoise/Grind	165 (93%)	0.50 (2.01)	0.0 (0.0-0.5)	4 (2.4%)	19 (86.4%)	13 (59.1%)	47 (27%)
Dried meat from narwhale and Beluga whale	172 (97%)	0.61 (1.51)	0.5 (0.0-0.5)	3 (1.7%)	36 (85.7%)	35 (83.3%)	59 (33%)
Dried meat from seal	170 (96%)	0.42 (1.15)	0.0 (0.0-0.5)	2 (1.2%)	29 (82.9%)	30 (85.7%)	46 (26%)
Dried meat from fin- and minke whale	173 (98%)	0.47 (0.80)	0.5 (0.0-0.5)	4 (2.3%)	31 (91.2%)	29 (85.3%)	59 (33%)
Seal blubber	173 (98%)	1.07 (2.11)	0.5 (0.0-1.0)	11 (6.4%)	45 (97.8%)	39 (84.8%)	77 (44%)
Whale blubber	169 (96%)	0.47 (0.79)	0.0 (0.0-0.5)	3 (1.8%)	27 (93.1%)	24 (82.8%)	62 (35%)
Narwhale blubber and mattak	169 (96%)	1.32 (2.65)	0.5 (0.5-1.0)	10 (5.9%)	63 (86.3%)	70 (95.9%)	86 (49%)
Suaatsat with seal or whale	169 (96%)	1.24 (1.89)	0.5 (0.5-1.0)	11 (6.5%)	58 (90.6%)	59 (92.2%)	96 (54%)
Walrus	166 (94%)	0.10 (0.28)	0.0 (0.0-0.0)	0 (0.0%)	9 (69.2%)	8 (61.5%)	26 (15%)
Polar bear	169 (96%)	0.03 (0.11)	0.0 (0.0-0.0)	0 (0.0%)	2 (100.0%)	2 (100.0%)	15 (8%)
Guillemot	163 (92%)	0.47 (0.59)	0.5 (0.0-0.5)	2 (1.2%)	18 (28.6%)	60 (95.2%)	62 (35%)
Common eider	168 (95%)	0.34 (0.60)	0.0 (0.0-0.5)	2 (1.2%)	15 (39.5%)	36 (94.7%)	52 (29%)
Kittiwake	162 (92%)	0.14 (0.31)	0.0 (0.0-0.0)	0 (0.0%)	10 (50.0%)	14 (70.0%)	33 (19%)
Other seabirds	176 (99%)	0.05 (0.22)	0.0 (0.0-0.0)	0 (0.0%)	1 (20.0%)	5 (100.0%)	8 (5 %)
Trout	168 (95%)	1.22 (1.83)	0.5 (0.5-1.0)	7 (4.2%)	79 (90.8%)	44 (50.6%)	77 (44%)
Cod	174 (98%)	1.24 (1.58)	0.5 (0.5-1.0)	6 (3.4%)	69 (97.2%)	61 (86.9%)	94 (53%)
Greenlandic halibut	175 (99%)	0.93 (0.93)	0.5 (0.5-1.0)	6 (3.4%)	62 (89.9%)	68 (98.6%)	88 (50%)
Atlantic halibut	171 (97%)	0.36 (0.58)	0.5 (0.0-0.5)	1 (0.6%)	27 (87.1%)	28 (90.3%)	61 (34%)
Redfish	173 (98%)	0.39 (0.54)	0.5 (0.0-0.5)	1 (0.6%)	38 (90.5%)	34 (81.0%)	60 (34%)
Salmon	173 (98%)	0.92 (1.55)	0.5 (0.5-1.0)	5 (2.9%)	54 (85.7%)	57 (90.5%)	83 (47%)
Wolfish	172 (97%)	0.32 (0.43)	0.0 (0.0-0.5)	0 (0.0%)	27 (87.1%)	24 (77.4%)	59 (33%)
Capelin	171 (97%)	0.69 (0.90)	0.5 (0.0-0.5)	4 (2.3%)	60 (96.8%)	37 (59.7%)	69 (39%)
Shrimp	176 (99%)	1.98 (4.37)	1.0 (0.5-2.5)	13 (7.4%)	71 (97.3%)	72 (98.6%)	93 (53%)
Clamp	172 (97%)	0.54 (0.70)	0.5 (0.0-0.5)	2 (1.2%)	43 (95.6%)	36 (80.0%)	69 (39%)
Crab	175 (99%)	0.75 (0.79)	0.5 (0.5-1.0)	2 (1.2%)	52 (89.7%)	56 (96.6%)	87 (49%)
Dried Greenlandic halibut	174 (98%)	0.63 (0.61)	0.5 (0.5-0.5)	1 (0.6%)	48 (87.3%)	55 (100.0%)	96 (54%)
Dried capelin	173 (98%)	0.93 (1.57)	0.5 (0.5-1.0)	4 (2.3%)	52 (98.1%)	47 (88.7 %)	84 (47%)
Dried cod	176 (99%)	1.97 (3.8)	1.0 (0.5-2.5)	18 (10.2%)	62 (92.5%)	65 (97.0%)	93 (53%)
Dried wolfish	172 (97%)	0.43 (2.34)	0.0 (0.0-0.5)	1 (0.6%)	20 (95.2%)	20 (95.2%)	47 (27%)
Dried other fish	177 (100%)	0.14 (0.45)	0.0 (0.0-0.0)	1 (0.6%)	5 (71.4%)	6 (85.7%)	24 (14%)
Grouse	169 (96%)	0.20 (0.42)	0.0 (0.0-0.5)	0 (0.0%)	12 (42.9%)	25 (89.3%)	29 (16%)

Greenlandic Lamb/Sheep	175 (99%)	1.57 (3.01)	1.0 (0.5-1.0)	13 (7.4%)	56 (96.6%)	57 (98.3%)	100 (56%)
Caribou	172 (97%)	2.75 (4.15)	2.5 (1.0-2.5)	25 (14.5%)	72 (87.8%)	77 (93.9%)	92 (52%)
Musk ox	174 (98%)	1.07 (2.72)	0.5 (0.5-1.0)	6 (3.4%)	51 (86.4%)	54 (91.5%)	78 (44%)
Hare	171 (97%)	0.13 (0.39)	0.0 (0.0-0.0)	1 (0.6%)	11 (64.7%)	15 (88.2%)	22 (12%)
Berries	163 (92%)	2.09 (3.70)	1.0 (0.5-2.5)	27 (16.6%)	65 (85.5%)	55 (72.4%)	89 (50%)
**Imported food items**							
Chicken/Turkey	177 (100%)	3.74 (3.92)	2.5 (1.0-4.3)	73 (41.2%)	72 (100.0%)	72 (100.0%)	103 (58%)
Pork	176 (99%)	5.18 (5.46)	2.5 (2.5-4.3)	87 (49.4%)	73 (100.0%)	72 (98.6%)	101 (56%)
Beef	177 (100%)	7.61 (5.77)	4.3 (2.5-13)	123 (69.5%)	71 (100.0%)	71 (100.0%)	105 (59%)
Imported Lamb/Sheep	174 (98%)	1.06 (1.68)	0.5 (0.5-1.0)	11 (6.3%)	53 (96.4%)	55 (100.0%)	83 (47%)
Imported fish	172 (97%)	1.08 (2.38)	0.5 (0.0-0.8)	17 (9.9%)	42 (97.7%)	41 (95.3%)	56 (32%)
Potato	176 (99%)	14.21 (10.17)	13.0 (4.3-13.0)	152 (86.4%)	68 (100.0%)	68 (100.0%)	107 (60%)
Pasta	176 (99%)	10.89 (8.40)	13.0 (4.3-13.0)	145 (82.4%)	69 (100.0%)	69 (100.0%)	107 (60%)
Rice	175 (99%)	11.19 (8.32)	13.0 (4.3-13.0)	145 (82.9%)	69 (100.0%)	69 (100.0%)	107 (60%)
Sauce	176 (99%)	16.51 (11.64)	13.0 (4.3-30.4)	148 (84.1%)	69 (100.0%)	69 (100.0%)	106 (60%)
Vegetables	177 (100%)	16.13 (12.30)	13.0 (4.3-30.4)	148 (83.6%)	68 (100.0%)	68 (100.0%)	105 (59%)
Fresh fruit	176 (99%)	15.45 (13.92)	13.0 (4.3-30.4)	140 (79.5%)	68 (100.0%)	68 (100.0%)	106 (60%)
Canned fruit	173 (98%)	1.02 (1.64)	0.5 (0.5-1.0)	10 (5.8%)	55 (96.5%)	57 (100.0%)	92 (52%)
Frozen fruit	175 (99%)	3.55 (7.04)	1.0 (0.5-2.5)	36 (20.6%)	54 (100.0%)	54 (100.0%)	95 (54%)
Dried fruit	175 (99%)	3.54 (7.53)	1.0 (0.5-2.5)	33 (18.9%)	58 (100.0%)	58 (100.0%)	92 (52%)
Pizza	176 (99%)	1.89 (3.64)	1.0 (0.5-2.5)	21 (11.9%)	71 (98.6%)	69 (95.8%)	97 (55%)
Ready-made meals	172 (97%)	1.35 (2.09)	0.5 (0.5-1.8)	11 (6.4%)	54 (100.0%)	54 (100.0%)	97 (55%)
Fast food and café-food	177 (100%)	2.26 (3.53)	1.0 (0.5-2.5)	28 (15.8%)	67 (100.0%)	67 (100.0%)	101 (57%)
Nuts	176 (99%)	5.02 (8.59)	2.5 (0.5-4.3)	53 (30.1%)	65 (100.0%)	65 (100.0%)	100 (56%)
Cake	176 (99%)	4.27 (6.30)	2.5 (1.0-4.3)	66 (37.5%)	66 (100.0%)	66 (100.0%)	109 (62%)
Milk chocolate	176 (99%)	4.44 (6.85)	2.5 (0.5-4.3)	62 (35.2%)	63 (100.0%)	63 (100.0%)	95 (54%)
Dark chocolate	177 (100%)	3.09 (5.97)	1.0 (0.5-4.3)	48 (27.1%)	56 (100.0%)	56 (100.0%)	91 (51%)
Candy	175 (99%)	7.72 (9.68)	4.3 (1.0-13.0)	94 (53.7%)	67 (100.0%)	67 (100.0%)	105 (59%)
Chips	174 (98%)	7.24 (8.70)	4.3 (2.5-13.0)	110 (63.2%)	67 (100.0%)	67 (100.0%)	110 (62%)

n (%): number of participants with information and percentages of the total number of participant (N); SD: Standard Deviation, P25-P75: 25 percentile – 75 percentile; [a] Number of participant reporting eating the food item season (Spring/Summer or Autumn/Winter), percentage in calculated based on the participants who gave seasonal information on the specific food item; [b] Missing information is calculated based on those who reported eating the food item, thus participants reporting never eating the food items is excluded from the calculations.
