# Supplementary material for: Dietary habits among men and women in West Greenland: follow-up on the ACCEPT birth cohort
Source: BMC Public Health. 2021 Jul 19;21:1426. doi: 10.1186/s12889-021-11359-7 (PMC8290613; doi:10.1186/s12889-021-11359-7)
Supplement: Supplementary file 3 — Additional file 3. [file 12889_2021_11359_MOESM3_ESM.pdf]

Additional file 3

Dietary habits among men and women in West Greenland: follow-up on the ACCEPT birth cohort

Age group differences in traditional and imported food intake (time(s) per month) stratified by gender

Table S3A: Traditional and imported food intake (time(s) per month) by age for the mothers (N=101)

	<34.0 years (n=50)			≥34.0 years (n=51)			p-value
	n (%)	Mean (SD)	Median (P25-P75)	n (%)	Mean (SD)	Median (P25-P75)	
**Traditional foods**			12 %[a]			15 %[a]	
Marine mammals	39 (78%)	7.70 (8.68)	5.5 (2.5-8.0)	37 (73%)	10.71 (12.01)	6.5 (4.5-11.0)	0.197
Seabirds	44 (88%)	0.75 (0.87)	0.5 (0.0-1.0)	45 (88%)	1.04 (1.45)	1.0 (0.0-1.5)	0.297
Greenlandic fish	46 (92%)	4.71 (3.82)	4.0 (2.0-5.5)	47 (92%)	7.82 (8.14)	6.0 (3.5-9.0)	**0.010***
Shellfish	49 (98%)	2.28 (1.78)	2.0 (1.0-3.5)	49 (96%)	2.93 (3.12)	2.0 (1.0-3.5)	0.475
Dried fish	48 (96%)	3.08 (2.79)	2.5 (1.5-4.3)	48 (94%)	5.42 (9.47)	2.5 (1.5-5.4)	0.337
Terrestrial animals	47 (94%)	4.56 (4.67)	3.5 (2.0-5.5)	47 (92%)	5.06 (5.78)	3.5 (2.0-5.5)	0.888
Berries	47 (94%)	3.17 (5.57)	1.0 (0.5-2.5)	48 (94%)	1.63 (2.15)	1.0 (0.5-1.8)	0.514
**Imported food**			88 %[a]			85 %[a]	
Meat products	47 (94%)	20.28 (9.90)	21.6 (10.3-29.5)	50 (98%)	18.15 (15.23)	13.9 (8.5-23.1)	0.066
Carbohydrate foods	48 (96%)	37.98 (19.17)	39.0 (27.8-39.0)	50 (98%)	36.24 (23.63)	39.0 (21.6-39.0)	0.587
Sauce	49 (98%)	16.36 (10.70)	13.0 (13.0-30.4)	51 (100%)	14.68 (10.76)	13.0 (4.3-30.4)	0.398
Vegetables	50 (100%)	18.24 (12.34)	13.0 (13.0-30.4)	51 (100%)	18.95 (12.55)	13.0 (13.0-30.4)	0.785
Fruits	49 (98%)	22.83 (19.27)	16.0 (5.3-32.4)	50 (98%)	25.54 (16.88)	17.9 (14.0-33.4)	0.228
Fast food	49 (98%)	4.81 (4.84)	3.0 (2.0-6.0)	49 (96%)	4.81 (6.09)	2.5 (1.5-6.0)	0.342
Sweets and Snacks	48 (96%)	37.25 (26.18)	29.7 (18.3-50.3)	49 (96%)	31.92 (17.36)	32.9(17.3-43.2)	0.547

Differences between age groups were tested with a Mann Whitney U test. Bold numbers and * indicate significant difference (p≤0.05). n (%): number of participants with information and percentages of the total number of participant in the group (N); SD: Standard Deviation; P25-P75: 25 percentile – 75 percentile; [a]The overall percentages of median intake of the main food groups, traditional (x) or imported food (y), were calculated by summing the medians of the main food groups and then the sum was divided by the total median intake (x+y).

Table S3B: Traditional and imported food intake (time(s) per month) by age for the fathers (N=76)

	<36.7 years (n=38)			≥36.7 years (n=38)			p-value
	n (%)	Mean (SD)	Median (P25-P75)	n (%)	Mean (SD)	Median (P25-P75)	
**Traditional foods**			15 %[a]			16 %[a]	
Marine mammals	25 (66%)	10.06 (13.48)	6.0 (2.5-9.0)	25 (66%)	8.02 (7.46)	5.5 (2.0-10.5)	0.801
Seabirds	33 (87%)	1.03 (1.02)	1.0 (0.5-1.5)	31 (82%)	0.98 (0.72)	1.0 (0.5-1.5)	0.767
Greenlandic fish	33 (87%)	5.62 (5.02)	4.5 (3.0-6.8)	34 (90%)	5.92 (3.92)	4.5 (3.5-7.5)	0.567
Shellfish	36 (95%)	3.13 (3.16)	2.5 (1.0-3.8)	36 (95%)	3.77 (5.04)	2.3 (1.5-4.3)	0.486
Dried fish	35 (92%)	4.06 (5.05)	2.5 (1.5-4.5)	37 (97%)	3.22 (2.15)	2.5 (1.5-4.5)	0.919
Terrestrial animals	33 (87%)	6.68 (8.99)	3.5 (2.5-6.0)	33 (87%)	7.80 (11.67)	4.5 (2.5-7.0)	0.733
Berries	34 (90%)	1.48 (2.37)	0.5 (0.5-1.0)	34 (90%)	1.86 (3.07)	0.8 (0.5-2.5)	0.651
**Imported food**			85 %[a]			84 %[a]	
Meat products	36 (95%)	18.07 (9.21)	17.3 (9.8-27.0)	37 (97%)	17.78 (12.64)	12.1 (8.0-30.8)	0.612
Carbohydrate foods	38 (100%)	34.74 (19.95)	37.2 (19.8-39.0)	37 (97%)	36.68 (26.59)	30.3 (26.5-39.0)	0.868
Sauce	38 (100%)	17.49 (11.80)	13.0 (4.3-30.4)	38 (100%)	18.17 (13.74)	13.0 (4.3-30.4)	0.970
Vegetables	38 (100%)	11.43 (11.05)	13.0 (2.5-13.0)	38 (100%)	14.25 (11.81)	13.0 (4.3-13.0)	0.205
Fruits	37 (97%)	9.66 (10.08)	5.8 (2.5-14.0)	36 (95%)	19.35 (17.77)	14.3 (5.3-32.9)	**0.015***
Fast food	35 (92%)	8.13 (8.8)	5.5 (2.5-9.1)	38 (100%)	5.09 (3.63)	4.4 (2.0-7.5)	0.147
Sweets and Snacks	37 (97%)	32.46 (26.24)	22.3 (10.5-52.6)	36 (95%)	33.12 (29.77)	23.5 (12.3-48.1)	0.952

Differences between age groups were tested with a Mann Whitney U test. Bold numbers and * indicate significant difference (p≤0.05). n (%): number of participants with information and percentages of the total number of participant in the group (N); SD: Standard Deviation, P25-P75: 25 percentile – 75 percentile; [a]The overall percentages of median intake of the main food groups, traditional (x) or imported food (y), were calculated by summing the medians of the main food groups and then the sum was divided by the total median intake (x+y).
